# Attitudes and Barriers to Breastfeeding among Mothers in Princess Nourah Bint Abdulrahman University, Riyadh, Kingdom of Saudi Arabia

**DOI:** 10.1155/2021/5585849

**Published:** 2021-07-29

**Authors:** Razan Yasser Abulreesh, Ibtihaj Abdullah Alqahtani, Zainah Yahya Alshehri, Maha Ali Alsubaie, Shatha Nasser Alburayh, Norah Mohammed Alzamil, Hayat Saleh Alzahrani

**Affiliations:** ^1^Faculty of Medicine, Princess Nourah Bint Abdulrahman University, Riyadh, Saudi Arabia; ^2^Family Medicine Department, Faculty of Medicine, Princess Nourah Bint Abdulrahman University, Riyadh, Saudi Arabia

## Abstract

**Background:**

Breastfeeding provides unsurpassed natural nutrition to the newborn and infant. It has a nearly perfect mix of food elements and vitamins that infants need to grow up. Nonetheless, the tendency for breastfeeding remains below the expected levels.

**Objectives:**

To explore the attitudes and barriers to breastfeeding among mothers in Princess Nourah Abdulrahman University (PNU), Riyadh, Saudi Arabia.

**Methods:**

A cross-sectional study was conducted, from January to April 2019; 399 PNU students, employees, and faculty mothers aged 18 years and above with experience of childbirth and breastfeeding were included in the study using a predesigned validated questionnaire. The questionnaire consisted of four scales: sociodemographic, attitude toward breastfeeding, barriers to breastfeeding, and induced lactation knowledge.

**Results:**

The participants' mean age was 34.1 ± 10.4 years; most (87.8%) were Saudi; 92.8% were married; 62% had a bachelor's degree; and 43% had “enough income.” While 40% of the mothers reported >6 months “exclusive breastfeeding” for the first baby, only 34.8% did so for the last baby, and 54.5% did so for most of all babies altogether. The mothers' parity ranged between 1 birth and 4 births in 23.5% and 17.5% of the participants, respectively. An overall score of breastfeeding attitude averaged 59.6 ± 7.3. The tendency for scoring a negative attitude to breastfeeding was significantly reported (*p* < 0.5) among 127 (31.8%) 31- to 40-year-old mothers; 153 (38.3%) bachelor's degree holders; and 157 (39.3%) employees (*χ*^2^ (4) 14.6, *p* = 0.006; *χ*^2^ (4) 10.4, *p* = 0.034; and *χ*^2^ (4) 20.4, *p* < 0.001, respectively). “Mother's illness” was the most commonly (63%) reported barrier to “not to breastfeed,” followed by “work” (45.5%) and “father not supporting breastfeeding” (14.8%).

**Conclusions:**

An overall negative attitude toward breastfeeding among PNU mothers was noted. Barriers included mother's sickness and work. Efforts to minimize such negative attitudes and barriers among susceptible mothers are warranted.

## 1. Introduction

Breastfeeding provides unsurpassed natural nutrition to the newborn and infant [1]. It has a nearly perfect mix of food elements and vitamins that infants need to grow up. Nonetheless, the tendency for breastfeeding remains below the expected levels that is why the World Health Organization (WHO) has set a goal of increasing the rate of exclusive breastfeeding to at least 50% by 2025 [2]. The benefits of breastfeeding in providing unique health and development opportunities for the infant are uncountable. Colostrum, which is secreted in the early hours following delivery, contains high levels of proteins, fats, and antibodies [3] together with leukocytes most of which are neutrophils and phagocytic macrophages that survive passage through the gastrointestinal system where they are absorbed and enforce the infant's immune response [1, 4].

Mature human breast milk then contains large quantities of secretory immunoglobulin A to line the respiratory and gastrointestinal mucosal membranes, rendering the infant four times less likely to develop diarrheal disease than formula-fed babies [5]. Such diarrheal disease is of shorter duration and is less severe than in formula-fed babies [6]. Likewise, breastfed infants exclusively for the first 6 months have a lower tendency for ear infections [7] and respiratory tract infections [8]. Compared to formula-fed infants, breastfed infants are more likely to gain the right amount of weight as they grow rather than become overweight children [9]. Further quality studies show that breastfeeding is associated with a significantly decreased incidence of type 2 diabetes and systolic blood pressure elevation later in life [10]. Evidently, infants who are breastfed exclusively for 6 months or longer compared with a shorter duration or not breastfeeding at all had a 20% lower risk for childhood leukemia [11].

The early feeding interactions, physical closeness, skin-to-skin touching, and eye contact between mother and infant enhance maternal-infant bonding and result in more positive feeding experience and produce greater maternal sensitivity and responsiveness to infant needs [12].

The production of prolactin and oxytocin during breastfeeding is associated with lower levels of maternal stress and enhanced bonding [13]. On contrary, early cessation of breastfeeding or not breastfeeding at all has been linked to an increased risk of maternal postpartum depression [14]. Lactating women have been found at a lower risk for developing premenopausal breast and ovarian cancer [15]. On the child's part, too, the cognitive development is significantly enhanced because of the rich vital nutrients in breast milk, such as essential fatty acids, vitamins, minerals, and amino acids [16]. Language development [17] and overall neurological development are largely enhanced [13, 18]. For all these reasons, the WHO recommends that breastfeeding be initiated within one hour of birth that it continues with no other foods or liquids for the first six months of life and continued with other age-appropriate foods until at least 24 months of age [19].

Attitudes and confidence among women can predict the duration of exclusive breastfeeding; the longer the duration of breastfeeding, the more the advantages for both mother and child. On the other hand, barriers may be linked to problems related to either the mother, the infant, or the breastfeeding technique [20, 21]. For instance, breast and nipple diseases as well as lactation complications, lack of self-efficacy, and limited social support may prevent mothers from lactating. Embarrassment toward breastfeeding in public together with an unsupportive childcare environment is a common cause of unfavorable breastfeeding practices [22, 23]. Maternal fatigue, stress, and other emotional problems are common health factors hindering exclusive breastfeeding. Mother's work particularly plays an important role in breastfeeding attitude. For instance, women planning to work full-time postpartum were less likely to initiate breastfeeding than women who planned to work part-time, and women were more likely to cease breastfeeding the first month prior or subsequent to returning to work [24]. Breastfeeding initiation and duration are reportedly lower among low-income women and women with lower education levels [25]. On the infant's part, being physically compromised or sustaining systemic illnesses interfere with the baby's ability to breastfeed successfully [20]. As a result of these and other problems, failure of initiation or discontinuity of breastfeeding is likely to occur.

In Saudi Arabia, there has been reluctance in practicing breastfeeding [26]; for example, only around 33% of mothers exclusively breastfeed their infants for the first four months [3]. Mixing formula with breastfeeding, instead, is a popular infant feeding choice in Saudi Arabia [26, 27]. To date, there is no national database on breastfeeding in Saudi Arabia [26]. Furthermore, there is little research about barriers to breastfeeding in the country. This study explored attitudes toward breastfeeding and associated barriers and evaluated the knowledge about induction of lactation among mothers in PNU in Riyadh.

## 2. Methods

### 2.1. Study Design, Setting, and Study Population

A cross-sectional design was selected for the study. The study was conducted in PNU in Riyadh, the capital of the Kingdom of Saudi Arabia (KSA), during the period between January 2019 and April 2019. Undergraduate students, employees, and faculty mothers aged 18 years and above with experience of childbirth and breastfeeding were included in the study. No PNU mother would be excluded from the study because of nationality, current marital status, occupation, or underlying health condition.

### 2.2. Study Sampling

Epi Info version 3 was used to calculate sample size “*n*,” using sample size for “frequency in a proportion” formula ((*n* = DEFF^*∗*^*Np* (1 − *p*))/((*d*^2^/*Z*^2^_1−*α*/2_(*N* − 1)+*p*^*∗*^(1 − *p*))), where population size (for finite population correction factor (fpc)) (*N*) = 250,000; *p* = hypothesized percentage frequency of outcome factor in the population = 50% ± 5 (to get maximum “*n*”); *d* = confidence limits as percentage of 100 (absolute +/− %) = 5%; and design effect (DEFF) (for cluster surveys) = 1 [28]. Our “*n*” equated 384, which was raised up to 412 to compensate for withdrawals and invalid responses. All mothers who agreed to participate were included in the study until the required sample size has been collected. The nature of the study population frame, like all were confined to PNU mothers, was not in favor of randomization; instead, a rather convenience sampling technique in recruiting the study participants' sample was adopted.

A validated self-administered questionnaire was developed to collect study data. The questionnaire consisted of four scales: sociodemographic, attitude toward breastfeeding, barriers to breastfeeding, and induced lactation knowledge. Sociodemographic scale included items (questions) relevant to breastfeeding, such as maternal age (years), nationality, marital status, level of education, occupation, living, income (all sources, all family members), payment, and number of live births. The breastfeeding attitude scale included items modified from the “Iowa infant feeding attitude scale” (IIFAS) [29]; IIFAS demonstrates high validity and reliability [30] and is widely utilized in the domain of measuring maternal attitudes toward infant feeding methods. The IIFAS consists of 17 items: 9 to reflect the positive attitudes to breastfeeding and 8 to reflect the negative ones (in favor of formula feeding). Items are scored on 5-point Likert-type scales, such as “strongly disagree,” “disagree,” “neutral,” “agree,” and “strongly agree”. Items that favor breastfeeding are scored positively, so that “strongly agree” is given a maximum score of 5, and a “strongly disagree” is given the least score of 1. Items in favor of formula feeding are scored vice versa. An overall attitude score is computed via the equal-weighted sum of responses to all items, so that the total score ranges from 17 to 85, with higher scores indicating positive attitudes to breastfeeding. Three main cutoff scores were set for our IIFAS: 17–60, 61–75, and 76–85, delineating negative, neutral, and positive attitudes to breastfeeding, respectively [31, 32].

An Arabic scale translated from the original English IIFAS version was prepared, with utmost attention to ensure proper retention of the content and semantic and technical equivalence of this version. In order to ensure that the scale measures the same words and concepts, back translation of the Arabic scale as a second language to the English version as the first language was carried out. In developing the scale on barriers to breastfeeding, the investigators first examined a body of literature on relevant topics [22, 33–36].

No standardized questionnaire with barriers to breastfeeding was available to advocate in the current study; therefore, we wanted to set items to address concepts pertinent to barriers toward breastfeeding, as concluded from the reviewed literature studies. For instance, principal “barriers” domains included: health status; breast condition, milk production, and impact of breastfeeding on breast shape; workplace and physical environment; cultural concerns, antenatal support, and postpartum support; emotional perception; and baby health issues. Accordingly, items identifying these concepts, including factors that encourage or discourage breastfeeding, have been phrased. Given that sensitive perception by many women to the inquiry about breastfeeding, anonymous inquiries were considered to allow mothers to answer more honestly and in a fast, efficient manner. Where appropriate, questions offered multiple-choice responses, so as the respondent could check more than one response [22].

Lastly, a scale on induction of lactation to integrate into the final questionnaire was also administered. Five items were introduced to identify the participants' opinion about relevant concepts, such as the possibility of inducing breast milk production, knowledge of ways to induce lactation, whether unmarried women are able to induce lactation, the possibility of reinducing lactation in postmenopausal women, and the possibility that woman who had never previously lactated can lactate successfully [37]. The final questionnaire was given to three consultants in the field: a pediatrician, a nutritionist, and a community health research scholar with an interest in maternal and child health promotion. The consultants were humbly asked to review and comprehensively evaluate the instrument in terms of construct and content validity. Refinement of the questionnaire draft was then done in response to the reviewers' notes and suggestions.

A pilot was conducted to assess the questionnaire's reliability. Twenty-five PNU mothers were given the questionnaire to respond (response-a). The same individuals were given the same questionnaire to readminister the questionnaire a week later (response-b). A panel of juries was selected to judge the responses; test-retest reliability was calculated to assess its temporal stability. Spearman's *rho* for each pair of items (response-a and response-b) was significant (*p*<0.05) for the examined items, with moderate–strong correlation (*rho* 0.79–0.90). Modifications of the questionnaire items were carried out based on the pilot testing results. The piloted subjects were not included in the study. Most of the study items represent qualitative data, including nominal/dichotomous data, e.g., nationality, profession, barriers to breastfeeding (yes or no); discrete data, e.g., number of births; or ordinal data, e.g., education level, income stratum, number of times breastfed, and breastfeeding duration stratum.

### 2.3. Ethical Considerations

First, an application for ethical approval to conduct the study has been submitted to PNU Institutional Review Board (IRB) before commencing the study. The study has been approved by the IRB. Invited women were briefed of the study type and aim. Verbal consent was considered an agreement to participate in the study. A paper-and-pencil questionnaire form was given to participants on the PNU campus for their response. The questionnaire takes around 20 minutes to complete. Mothers were assured that their participation was voluntary and that they could opt out of the study at any time without giving reasons. They were further reassured of the confidentiality of the provided information and that only anonymous data would be disseminated. The returned questionnaires were coded, including data entered to a Microsoft program, with adequate backups until analyzed. Returned questionnaires with ≥80% valid answers would be entered into the analysis.

### 2.4. Statistical Analysis

Descriptive statistics in terms of count (%) and the means ± standard deviations (SD) or the median and interquartile ranges (IQR), where appropriate, would be used to describe the characteristics of the studied sample. In the inferential round of statistics, the strength of association between qualitative variables would be analyzed by the chi-square test of independence (or Fisher's exact test, where appropriate). Our alpha level to reject a true null hypothesis was *α* = 0.05, and the results with a *p* values <0.05 were considered statistically significant.

## 3. Results

As in Table 1, 49.8% of the mothers aged between 31 and 40, with mean age 34.1 ± 10.4 years (Table 1, footnote). Also, 87.8% were Saudi; 92.8% were married; 62.5% had a bachelor's degree; and most (61.8%) were employees. Living-wise, 40.5% of the mothers owned their house, and also, most of them (43.0%) had enough income. Parity-wise, the frequency of having either one or two babies was almost equal (23.5% and 22.5, respectively) (Table 1).

The first baby tended to be exclusively breastfed for >6 months more frequently (40%) than the other 2 breastfeeding durations (36% for 1–5 months and 24% for <1 month) (Table 2). In contrast, the last baby was breastfed more frequently for <1 month, compared with the other 2 breastfeeding durations (34.8% for >6 months and 24.2% for < 1 month). As in the first baby trend, most of all babies were breastfeed most reportedly (54.5%) for >6 months, 30% for 1–5 months, and least reportedly (15.5%) for < 1 month (Table 2).

The majority of the mothers reported that sickness was the largest barrier that prevented them from breastfeeding their baby (63%), and the second barrier was work (46.5%). Lack of encouragement from fathers was found to be the least common barrier toward breastfeeding (14.8%) (Table 3).

Table 4 displays attitudinal responses toward breastfeeding questions. Mothers were generally negative toward breastfeeding (mean total score = 59.6 ± 7.3). The mean score was not significantly different from the cutoff limit of 60 for negative attitudes against breastfeeding (*t*(df 399) = −1.37; *p*=0.17) (Table 4 footnotes). The majority of responses on most of the 8 questions that reflect negative attitudes to breastfeeding were unfavorable. For instance, 117 (29.2%) agreed that the “benefits of breast milk last only until weaning” (question 1, mean score = 2.9 ± 1.4) (Table 4). Likewise, as many as 183 (45.8%) agreed that “formula feeding was the better choice to plan to go out to work” (question 6, mean score = 2.4 ± 1.1); 93 (23.2%) agreed that “women should not breastfeed in places” (question 8, mean score = 2.9 ± 1.3); and 108 (27.0%) believed that “breastfed babies are more liable to overfeeding” (question 10, mean score = 3 ± 1).

In Table 5, three main attitudinal categories to breastfeeding are shown: negative, neutral, and positive, in relationship with ten sociodemographic characteristics examined. Only four (age, level of education, profession, and healthcare payment method) characteristics showed a significant influence upon breastfeeding (*χ*^2^ (4) = 14.6, *p*=0.006; *χ*^2^ (4) = 10.4, *p*=0.034; *χ*^2^ (4) = 20.4, *p* < 0.001; and *χ*^2^ (4) = 12.2, *p*=0.016, respectively). First, an overall 223 (55.8%) participants scored a negative attitude, 171 (42.7%) participants were neutral, and only 6 (1.5%) were positive to breastfeeding (Table 5 footnote). In 3 out of the 4 significant analyses, the most frequently occurring observation was that for the negative attitude and other attitudes in the same sociodemographic comparison, e.g., 127 (63.8% of row and 31.8% of total 400) of 31–40 years old. Also, 153 (51.2% of row and 38.3% of total) of bachelor holders and 157 (63.6% of row and 39.3% of total) of employees expressed a negative attitude to breastfeeding. On the other hand, postgraduate educated mothers reacted more favorably to breastfeeding that 45/83 (54.2%) were neutral, while 36/83 (43.4%) were negative to breastfeeding. Likewise, 43/71 (60.6%) of faculty members were neutral to breastfeeding, compared to 86/247 (3 4.8%) of employees and 42/82 (51.2%) of students. In the health care payment type comparison, 103 (49.5% of row and 25.8% of total) of the governmentally insured were neutral, followed by 100 (48.1% of row and 25% of total) of the same insurance category who were negative to breastfeeding. Income, the number of births, and the number of times mothers ever breastfed did not influence the attitude to breastfeeding (*p* < 0.05 all analyses).

A total of 73.5% of participants agreed with the concept of “induced lactation” (Table 6). The “most known way to increase milk production” was “drugs and herbal remedies”. Otherwise, 14.2% of the participants were uncertain about “the ability of premenopausal women to induce lactation. Only 9% of the participants were against reinducing lactation in nonlactating females (Table 6 footnote).

## 4. Discussion

Most infants today still do not receive the full benefits of breastfeeding, leaving millions at unnecessary risk of illness and death, and most health workers lack the skills needed to help mothers improve their feeding practices [38]. The problem involves a multitude of attitudinal issues besides issues that pose barriers to exclusive breastfeeding. Most of our participants' responses to those questions addressing negative attitudes to breastfeeding were unfavorable. As such, 55.8% of PNU mothers responded negatively, and 42.7% were neutral to breastfeeding, all with a mean score (59.6) that did not significantly differ from the study instrument's cutoff score of 60. In Riyadh, the study by Saied et al. [3] showed that the majority (89.4%) of mothers had a neutral attitude toward breastfeeding. The more likelihood of a negative attitude to breastfeeding in our study may be attributed to the situation that most (61.8%) of our participants were working mothers, while 63.1% of Riyadh's mothers were housewives.

Among our findings, age less than 40 was more likely met with a negative attitude to breastfeeding. Comparably, Laanterä et al. found that women below 27 did not tend to initiate breastfeeding [39]. In this work, Saudi mothers did not differ in their attitude to breastfeeding with peers of other nationalities, for example, contrary to what had been found when Saudis were compared with Egyptian peers, where only 14% of Saudi mothers reacted negatively to exclusive breastfeeding versus 65% of Egyptian mothers who behaved otherwise [26].

Mothers who were living in their workplace residency had a more positive attitude (53.3%) due to the close proximity, a finding which was also in line with that reported by other studies [40].

Pooled data analysis of a large population-based study from Nepal revealed that maternal education was consistently associated with a higher likelihood of early initiation of breastfeeding [41]. In our study, only postgraduate education and being academia were associated with a neutral attitude, rather than a negative attitude to breastfeeding.

The primary barrier preventing the initiation of breastfeeding among PNU mothers was sickness. Likewise, mothers' sickness was the second most commonly given reason for discontinuation of breastfeeding among mothers in Riyadh, KSA [42]. Work commitment was the second barrier in our study (46.5%). In fact, the mother's work has been consistently a significant barrier to all aspects of breastfeeding practice. For instance, Kimbro [24] found that the timings a lactating mother quits breastfeeding and that when she returns to work were closely linked, and that mothers in administrative and manual positions tended to quit earlier than other women. Dunn et al. [22] reported that breastfeeding duration significantly correlated with the mother's employment status and that among women who breastfed for 6 months or longer, only 15% were employed full-time, 30% worked part-time, and 55% were not committed to work. Likewise, Lauer et al. [43] found that women in specific service-oriented industries (accommodation and retail) reported the lowest rates of breastfeeding initiation and workplace supports for breastfeeding and pumping.

In our study, 39% of mothers were embarrassed about breastfeeding their babies in public places. In a study to reveal barriers to breastfeeding in Kuwait [44], most mothers reported feeling embarrassed to practice breastfeeding in public, e.g., 85.5% felt embarrassed to pump their breasts at work, 74.9% felt shy about breastfeeding outside the home, and 60.7% feared that people would see their breasts. Likewise, a study in Mississippi, USA, showed similar results of a sense of embarrassment, specifically about pumping breasts at school or work, breastfeeding outside their homes, and breastfeeding in front of family members [45]. In this work, too, mothers who indicated that the father does not encourage breastfeeding accounted for the least proportion among barriers to breastfeeding (14.8%). In Kuwait, too, among the members of social support, the more frequently mentioned persons were the husbands (84.7%) [44]. In the Mississippi study [45], time and social constraints presented barriers to fewer women than the embarrassment issues. In the present study, the embarrassment barrier was the most common, and lack of social support was perceived as the least common barrier.

Medical advances now developed techniques facilitating induced lactation and allowing nulliparous mothers the ability to breastfeed their children [45]. The present study demonstrated that only 9% and 14% of participants agreed that women can breastfeed at any age and that lactation can be induced in nonlactating women, respectively, indicating a knowledge gap about induced lactation. These findings were similar to what have been found in Malaysia, where also herbal medication was the most commonly known method to increase breast milk production [46].

### 4.1. Strengths and Limitations

The evidence-based data collecting instrument enabled identifying and measuring aspects of breastfeeding behavior and correlates the study aimed to examine, ensuring a level of validity of the achieved data. The relatively large sample size as well as the high test-retest and reproducibility of the developed questionnaire assure adequate generalizability of the obtained results in the general population setting.

The chi-square test may not show the optimal results in consideration of the low number of subjects, for example, the marital status and nationality (Table 5).

On the other hand, selecting the study sample in professional strata as exactly proportionate as those of PNU mothers' population would have been impractical; the reason why utilizing a convenience sampling technique in recruiting the calculated sample size was an alternative.

In addition, information regarding the benefits of breastfeeding was provided along with participants' questions were discussed and answered.

## 5. Conclusions

This research demonstrates a predominance of a negative attitude toward breastfeeding among PNU mothers. Significant relationships are noted between this attitude and age, nationality, education, profession, payment, and living. Older, more highly educated, and non-Saudi mothers had more positive attitudes toward breastfeeding. In contrast, the relationship between income, number of births, and number of times breastfeeding was less significant. Mother's illness and work represented barriers with the highest percentages among reasons preventing breastfeeding, and several other barriers are also noted. There is good information about how to increase the production of milk, yet information about the induction of lactation was limited.

### 5.1. Recommendations

With respect to the obtained findings that were less likely in favor of breastfeeding and the associated determinants, particular recommendations include: (a) providing health education programs in student curricula, (b) support the creation of a breastfeeding-friendly work environment, and (c) conducting awareness campaigns about induction of lactation. Further research to provide guidance regarding inducing lactation in Saudi Arabia is suggested.

## Figures and Tables

**Table 1:** Distribution of participants by sociodemographic characteristics (*n* = 400).

Category	Subcategory	*n* (%)
Age (years)^*∗*^	20–30	118 (29.5)
	31–40	199 (49.8)
	>40	83 (20.8)

Nationality	Saudi	351 (87.8)
	Non-Saudi	49 (12.2)

Marital status	Married	371 (92.8)
	Widow/divorced	29 (7.2)

Level of education	Before college	67 (16.8)
	Bachelor's degree	250 (62.5)
	Postgraduate	83 (20.8)

Profession	Student	82 (20.5)
	Employee	247 (61.8)
	Faculty member	71 (17.8)

Living	Own house	162 (40.5)
	Rent	148 (37.0)
	Large house	45 (11.2)
	Workplace	45 (11.2)

Income (all sources, all family members)	Not enough but I borrow	20 (5.0)
	Enough	172 (43.0)
	Not enough	62 (15.5)
	Enough and save	97 (24.2)
	I don't want to answer	49 (12.2)

Payment	Governmental	208 (52.0)
	Private	103 (25.8)
	Health insurance	89 (22.0)

Number of births	One time	94 (23.5)
	Two times	90 (22.5)
	Three times	74 (18.5)
	Four times	70 (17.5)
	>Four times	72 (18.8)

^*∗*^Mean age = 34.1 ± 10.4.

**Table 2:** Distribution of the study participants by the duration of exclusive breastfeeding (*n* = 400).

Child order	Breastfeeding duration	*n* (%)
First baby	Less than one month	96 (24.0)
	1–5 months	144 (36.0)
	More than 6 months	160 (40.0)

Last baby	Less than one month	164 (41.0)
	1–5 months	97 (24.2)
	More than 6 months	139 (34.8)

Most of all babies	Less than one month	62 (15.5)
	1–5 months	120 (30.0)
	More than 6 months	218 (54.5)

**Table 3:** Distribution of the study group by barriers to breastfeeding (*n* = 400).

#	Barrier	*n* (%)
1	Sick	252 (63.0)
2	Work	186 (46.5)
3	Tired	174 (43.5)
4	Taking contraceptives	165 (41.2)
5	Disease could transfer to the baby through breastfeeding	164 (41.0)
6	Too busy to breastfeed the baby	163 (40.8)
7	Embarrassed from lactation in public places	156 (39.0)
8	Perception of insufficient milk production	129 (32.2)
9	Pain presents an obstacle to breastfeeding	122 (30.5)
10	Depressed because my child refused breastfeeding	114 (28.5)
11	Poor prenatal and postpartum support	105 (26.2)
12	Unpleasant odor in the nursing mom	81 (20.2)
13	Housekeeper availability encourages me to provide bottle-feeding	79 (19.8)
14	I don't have enough knowledge	67 (16.8)
15	Fear of distorted breast shape by breastfeeding	61 (15.2)
16	Father does not encourage breastfeeding	59 (14.8)

**Table 4:** Itemized scoring of the mothers' attitudes to breastfeeding.

Attitude toward breastfeeding		Strongly disagree		Disagree		Neutral		Agree		Strongly agree		Mean^*∗*^ ± SD
		*N*	%	*n*	%	*n*	%	*n*	%	*n*	%	
Q 1	Benefits of breast milk last only until weaning	64	16.0	90	22.5	63	15.8	117	29.2	66	16.5	2.9 ± 1.4
Q 2	Formula milk is more convenient than breast milk	62	15.5	106	26.5	97	24.2	102	25.5	33	8.2	3.1 ± 1.2
Q 3	Breastfeeding strengthens mother-infant bonding	10	2.5	9	2.2	22	5.5	116	29.0	243	60.8	4.4 ± 0.9
Q 4	Breast milk lacks iron	81	20.2	123	30.8	124	31.0	56	14.0	16	4.0	2.5 ± 1.0
Q 5	Formula-fed infants are more liable to overfeeding	16	4.0	64	16.0	133	33.2	143	35.8	44	11.0	3.3 ± 1.0
Q 6	Formula milk is the better choice to plan to go out to work	18	4.5	62	15.5	68	17.0	183	45.8	69	17.2	2.4 ± 1.1
Q 7	Mothers who formula feed miss one of the great joys of motherhood	13	3.2	32	8.0	62	15.5	159	39.7	134	33.5	4.0 ± 2.1
Q 8	Women should not breastfeed in places	54	13.5	92	23.0	83	20.8	93	23.2	78	19.5	2.9 ± 1.3
Q 9	Breastfed babies are healthier	54	13.5	92	23.0	83	20.8	93	23.2	78	19.5	4.0 ± 1.0
Q 10	Breastfed babies are more liable to overfeeding	20	5.0	65	16.2	155	38.8	108	27.0	52	13.0	3.0 ± 1.0
Q 11	Father feel left out if a mother breastfeeds	72	18.0	123	30.8	126	31.5	66	16.5	13	3.2	3.4 ± 1.0
Q 12	Breast milk is the ideal food for the baby	8	2.0	7	1.8	18	4.5	125	31.2	242	60.5	4.5 ± 0.8
Q 13	Breast milk is more easily digested	13	3.2	8	2.0	39	9.8	107	26.8	233	58.2	4.3 ± 0.9
Q 14	Formula is as healthy for infant as breast milk	134	33.5	160	40.0	72	18.0	25	6.2	9	2.2	4.0 ± 1.7
Q 15	Breastfeeding is more convenient than formulas	18	4.5	79	19.8	92	23.0	103	25.8	108	27.0	3.5 ± 1.2
Q 16	Breast milk is less expensive than formula	10	2.5	10	2.5	14	3.5	140	35.0	226	56.5	4.4 ± 0.9
Q 17	Occasionally smoking mothers should not breastfeed	33	8.2	38	9.5	99	24.8	115	28.8	115	28.8	2.4 ± 1.2

^*∗*^Participants' mean score = 59.6 ± 7.3; one sample *t*-test: *t* (df 399) = −1.37; *p*=0.17.

**Table 5:** Distribution of breastfeeding attitude by sociodemographic characteristics: age, nationality, marital status, education, profession, living, payment, and income.

Characteristic	Category	Attitude to breastfeeding^*∗*^								Test statistic	*p* value
		Negative (17–60)		Neutral (61–75)		Positive (76–88)		Total			
		*N*	%	*n*	%	*n*	%	*n*	%		
Age (years)	20–30	63	53.4	53	44.9	2	1.7	118	100	*χ*^2^ (4) = 14.6	0.006
	31–40	127	63.8	69	34.7	3	1.5	199	100		
	More than 40	33	39.8	49	59.0	1	1.2	83	100		

Nationality	Saudi	199	56.7	147	41.9	5	1.4	351	100	*χ*^2^ (1) = 1.1	0.58
	Non-Saudi	24	49.0	24	49.0	1	2.0	49	100		

Marital status	Married	206	55.5	159	42.9	6	1.6	371	100	*χ*^2^ (1) = 0.53	0.77
	Widow/divorced	17	58.6	12	41.4	0	0.0	29	100		

Level of education	Before college	34	50.7	33	49.3	0	0.0	67	100	*χ*^2^ (4) = 10.4	0.034
	Bachelor	153	61.2	93	37.2	4	1.6	250	100		
	Postgraduate	36	43.4	45	54.2	2	2.4	83	100		

Profession	Student	40	48.8	42	51.2	0	0.0	82	100	*χ*^2^ (4) = 20.4	<0.001
	Employee	157	63.6	86	34.8	4	1.6	247	100		
	Faculty member	26	36.6	43	60.6	2	2.8	71	100		

Living	Own house	92	56.8	67	41.4	3	1.9	162	100	*χ*^2^ (6) = 6.5	0.37
	Rent	90	60.8	57	38.5	1	0.7	148	100		
	Large house	22	48.9	22	48.9	1	2.2	45	100		
	Workplace	19	42.2	25	55.6	1	2.2	45	100		

Healthcare payment	Governmental	100	48.1	103	49.5	5	2.4	208	100	*χ*^2^ (4) = 12.2	0.016
	Private	68	66.0	35	34.0	0	0.0	103	100		
	Health insurance	55	61.8	33	37.1	1	1.1	89	100		

Income	Not enough; borrow	9	45.0	11	55.0	0	0.0	20	100	*χ*^2^ (8) = 4.4	0.82
	Enough	102	59.3	67	39.0	3	1.7	172	100		
	Not enough	35	56.5	26	41.9	1	1.6	62	100		
	Enough and save	49	50.5	46	47.4	2	2.1	97	100		
	Don't answer	28	57.1	21	42.9	0	0	49	100		

Number of births	One time	50	53.2	43	45.7	1	1.1	94	100	*χ*^2^ (8) = 6.2	0.62
	Two times	52	57.8	36	40.0	2	2.2	90	100		
	Three times	44	59.5	28	37.8	2	2.7	74	100		
	Four times	43	61.4	27	38.6	0	0	70	100		
	>Four times	34	47.2	37	51.4	1	1.4	72	100		

Number of breastfed babies	One baby	57	52.3	52	47.7	0	0	109	100	*χ*^2^ (8) = 15.2	0.0.55
	Two babies	66	58.4	45	39.8	2	1.8	113	100		
	Three babies	45	60.8	25	33.8	4	5.4	74	100		
	Four babies	33	56.9	25	43.1	0	0	58	100		
	>Four babies	22	47.8	24	52.2	0	0	46	100		

^*∗*^223 (55.8%) scored a negative attitude; 171 (%42.7) were neutral; and 6 (1.5%) scored a positive attitude.

**Table 6:** The participants' attitude toward induced lactation concept.

Group^*∗*^	Agree *n* (%)	Disagree *n* (%)	Total *n* (%)
Lactation can be indicted in married women	294 (73.5)	106 (26.5)	400 (100)
Lactation can be indicted in premenopausal women	57 (14.2)	343 (85.8)	400 (400)

^*∗*^All participants' attitudes to reinducing lactation in postmenopausal women: 91% agree and 9% disagree.

## Data Availability

All data associated with this study are present in the paper.
